# Personality Traits and Emotion Regulation Styles of Elite Beach Volleyball Dyads: Examination of Intra-Team Differences, Performance and Satisfaction Levels

**DOI:** 10.3389/fpsyg.2021.719572

**Published:** 2021-10-22

**Authors:** Stefanie Klatt, Lisa-Marie Rückel, Sebastian Wagener, Benjamin Noël

**Affiliations:** ^1^Institute of Exercise Training and Sport Informatics, German Sport University Cologne, Cologne, Germany; ^2^School of Sport and Health Sciences, University of Brighton, Brighton, United Kingdom

**Keywords:** volleyball association, dyads, performance, team composition, personality

## Abstract

The current study was designed to assess the personality traits and emotion regulation styles of elite beach volleyball players. Intra-team differences were examined with three primary objectives: (i) to create a personality profile of elite beach volleyball players, (ii) to examine the relationship of this profile in relation to performance and satisfaction levels, and (iii) to highlight the similarities in personalities of members of successful teams. A total of 82 elite beach volleyball players were asked to fill out the Big Five Inventory, the Personality Adjective Scale, and the Affective Style Questionnaire. In addition to these, the overall satisfaction and performance level of these athletes were measured. Results indicated a higher manifestation of warmth, liveliness, emotional stability and reasoning, along with lower levels of neuroticism in successful athletes. The players used a variety of emotional regulation styles and reported being moderately to highly satisfied with their team. A repeated-measures MANCOVA revealed no significant differences in personality traits between the team members. This study generates valuable insights into the personality of elite beach volleyball players and can be useful for coaches, sport psychologists, and academics for practical application and further scientific research.

## Introduction

Practitioners of sports psychology and academics often refer to the general definition of a social group given by Sherif (1956) in order to define a sports team. According to this definition, a social group is defined by the interaction of two or more individuals who are dependent on each other and share common motives, interests, norms, values, and goals (Sherif, 1956; Forsyth, 2014). When group members commit themselves to the group, it results in feelings of cohesion and unity (Sherif, 1956; Forsyth, 2014).

However, with regard to a sports team, age, gender, playing position, technique, performance, and physical as well as functional parameters are more relevant as unifying factors than sharing equal norms and values (Trninić et al., 2008; Dadelo et al., 2014; Budak et al., 2018). The primary goal of high-performing teams is to win competitions and an efficient team is characterized by coordinated actions and cognitions of the team members (Bourbousson et al., 2010; Santos et al., 2018). While the emphasis in sports mainly lies on performance, teams are, in fact, seen as social groups. Therefore, the overall influence of shared norms, values, and personality traits could also play an important role in the success of the team and lead to enhanced teamwork and intra-team coordination.

Sports administrators, scouts, and coaches may have to consider these aspects to identify individuals who can form sustainable, high-performance teams. During the second stage of team development (*storming*) especially, conflicts and personality clashes can occur which need to be resolved urgently for the team to be cohesive and perform at a high level (Tuckman, 1965). This phase is particularly crucial for teams of smaller sizes, e.g., beach volleyball teams. Research shows that a good relationship and unity between team members is of greater importance for small teams in comparison to larger team sizes (Carron and Spink, 1995). It is also noteworthy that enjoyment and cohesion tends to decrease with an increase in the team size (Widmeyer et al., 1990).

The smallest team size is a dyad or a pair which is composed of two individuals (Becker and Useem, 1942). A dyad might require a closer relationship between both team members to maintain harmony and enable better team functioning. Compared to bigger groups, dyads cannot be broken down into sub-groups and split up when one member leaves (Forsyth, 2014). Therefore, dyad members are strongly inter-dependent and often show deep emotional bonds (Levine and Moreland, 2012). It is this codependence which makes the role of individual personality traits, beliefs, and values in a dyad unclear.

The personality of an individual is defined as a stable set of characteristics that influence and shape cognition, emotions, and behavior of that individual in various situations (Ryckman, 2012). In order to better understand and predict human behavior, researchers have tried to place human personality within the constructs of scientific models. So far, several personality models and theories thereof, have been shaped by the evolution and development of the broader field of psychology and philosophy. Trait psychology theories are the most scientific, research-based approach of describing human personality (Ellis et al., 2009). Traits are aspects of personality that are stable over time and across various situations. They have a certain measurable range of manifestation and differ among individuals (Boyle et al., 2008; Ellis et al., 2009).

In the past, several models of trait personality have been developed for research into this subject area, beginning with Allport (1937), Cattell (1943, 1973) and Eysenck (1957). Cattell was the first to develop a scientific measurement of trait personality by the use of a statistical factor analysis. He identified 16 personality traits like reasoning, warmth, emotional stability, and openness to change as the basis for his 16 Personality Factor Questionnaire (16 PF; Cattell, 1943). Eysenck (1957, 1967) built on this approach; however, he only focused on the three dimensions of extraversion, neuroticism, and psychoticism. The last and so far the most accepted personality trait theory is the Five Factor Model (FFM) based on the work of Goldberg (1990) and Costa and McCrae (1992a). The authors hypothesized that personality can be described best by five factors: extraversion, neuroticism, openness to experience, agreeableness, and conscientiousness (for detailed information about the factors, see Costa and McCrae, 1992b).

For years, sports psychologists have focused on the relationship between individual personality characteristics and success. What has emerged is that certain personality traits have an influence not only on the performance and success of the individual athlete (Allen et al., 2013), but also of the whole team (Halfhill et al., 2005). A review by Allen et al. (2013) discovered that elite athletes have higher levels of extraversion, conscientiousness, and agreeableness and lower level of neuroticism. These findings were related to success, effective coping, and mental preparation (Woodman et al., 2010). Compared to individual sports athletes, team sports athletes appear to be more extraverted and less conscientious (Eagleton et al., 2007; Allen et al., 2011). In spite of these findings, scientific inquiry on optimal distribution of personality traits within teams remains limited (Allen et al., 2013; Berger et al., 2015).

In studies by Schurr et al. (1977) and Kirkcaldy (1982) differences in the personalities within the team have been examined. Results show that offensive players tend to be more extraverted than team members playing defense. However, these results have not been confirmed by recent research (Jackson et al., 2010, 2011). Cameron et al., 2012 found that dissimilarities in the personality traits of extraversion and openness between dyadic team partners were associated with a higher probability of conflicts and dysfunctional intra-team relationships.

Based on the findings by Jackson et al. (2010, 2011), it can be proposed that personality and characteristics, specifically, similar beliefs, norms, and values, of both players within a dyadic team might be a crucial aspect in team formation and sustenance. In order to achieve harmony, good intra-team relationships and efficiency leading to success, coaches or scouts may wish to consider building dyadic teams consisting of similar personalities (Budak et al., 2018). However, teams are sometimes more efficient and successful if team members, who differ in their personalities, have unique and diverse attributes (Beebe and Masterson, 2010; Gilley et al., 2010). Mohammed and Angell (2003) investigated the performance of and relationship between differences in personality traits across 267 business students in 59 working teams. They found that overall differences in agreeableness and neuroticism resulted in poorer performance, whereas high variability of extraversion led to better performance.

Therefore, with this study, we have tried to provide further information about the role of the personalities of elite beach volleyball players within teams. The goal of this study was threefold: (1) creation of a personality profile of elite beach volleyball players, (2) examination of the relationship with their performance and satisfaction and (3) observation of personality differences between the members of a dyadic team. As most existing research has only considered the Big Five Personality Traits, we additionally integrated the 16 personality factors of Cattell (1943). We also included emotion regulation styles into our research as it is strongly associated with personality and is an important factor in elite sport (Uphill et al., 2012; Allen and Laborde, 2014; Barańczuk, 2019). Based on initial research, we formulated the following hypotheses:

*Hypothesis 1*: Elite beach volleyball players exhibit more characteristics of extraversion, agreeableness, openness, and fewer characteristics of neuroticism.

*Hypothesis 2*: Performance of elite beach volleyball players has a positive correlation with the personality traits of extraversion, agreeableness and conscientiousness and a negative correlation to neuroticism.

*Hypothesis 3*: Elite beach volleyball teams show high level of similarity of personality characteristics within their team.

## Materials and Methods

For data collection, we used an online survey^1^ and provided it digitally to the Top 50 of the German Volleyball Association. We also approached regional associations and 16 of the best female and male regional beach volleyball teams provided data through a paper-pencil test.

### Participants

For this study, only beach volleyball teams were considered. As the organizational rules of beach volleyball do not allow a coach to stand on the sideline during a competition (see official rules from the Fédération Internationale de Volleyball [FIVB], 2016), the team members have to interact, change tactics, and solve problems together among themselves without any external help. We also considered that beach volleyball players, in contrast to doubles teams in other sports like badminton or tennis, need to pass each other the ball and therefore, are equally responsible for the success or failure of the team.

A total of 82 beach volleyball athletes participated in the study, which was carried out from July to September 2017. Of those, 46 were male and 36 were female. The mean age of the participants was 26.39 (*SD* = 4.32) years and 31 played the position of block (37.8%), 34 of defense (41.5%) and 17 played both the positions alternately (20.7%). The criteria for the selection of the subjects were: (1) they could name a standard beach volleyball partner who also participated in the study; (2) they participated actively in tournaments in the 2017 season; (3) they were at least 18 years old; (4) they had the experience of participating in national competitions (at least A^2^ level), and (5) they signed informed consent approved by the ethics committee of the German Sport University Cologne (ethics proposal number: 135). The questionnaire, which was sent to them, assessed demographic variables, emotional regulation, personality type, level of satisfaction, and ranking points, which provided us an estimate on the performance level of an athlete. The study was carried out in accordance with the ethical standards laid down in the 1964 Declaration of Helsinki and its later amendments.

### Measurements

Data about an athlete’s personality was collected using the German Big-Five-Inventory-10 (BFI-10; by Rammstedt and John, 2007) and the German Personality Adjective Scale (PASK-5; Persönlichkeits-Adjektiv-Skalen) by Brandstätter (2009), which is based on Cattell’s personality theory and questionnaire (Cattell, 1943; Cattell and Mead, 2008). The BFI-10 questionnaire consisted of 10 items that were rated on a 5-point Likert scale (1 = “disagree strongly”; 5 = “agree strongly”) with a higher number indicating a greater compliance to the described statement. Five items (1, 3, 4, 5, and 7) had to be recoded. The identified personality dimensions were extraversion, neuroticism, openness to experience, conscientiousness, and Agreeableness (internal consistency from α = 0.58 to α = 0.84).

For the PASK5, participants rated opposing adjectives placed at the end of a 9-point Likert scale (e.g., ‘reserved’ – ‘outgoing,’ ‘affected by feelings’ – ‘emotionally stable’; internal consistency from α = 0.53 to α = 0.88). We used the German version of the Affective Style Questionnaire (ASQ) from Graser et al. (2012; original version from Hofmann and Kashdan, 2010) for measuring the emotion regulation strategies in high-performance beach volleyball athletes. The self-report survey consisted of 20 items (e.g., ‘I can tolerate strong emotions,’ or ‘I can calm myself down easily’) that were ranked on a 5-point Likert scale (1 = “not true for me at all”; 5 = “extremely true for me”) and averaged into one of the three regulation styles: Concealing (eight items), Adjusting (seven items), and Tolerating (five items). The ASQ is known to be a reliable and valid psychological measurement (internal consistency: suppression scale: α = 0.84; adjusting scale: α = 0.75; accepting scale: α = 0.72 Graser et al., 2012).

Furthermore, we used the individual ranking points as an estimation for performance level. Ranking points were amassed by the athletes by competing in tournaments where both the team members earn points individually for their performance and placements on the ranking scale. The higher final placements in the competition lead to more ranking points. For example, when finishing third in a competition, both team members gain 75 points, but members of the team placed first, earn 100 points each^3^. The points system also differs when playing against elite or professional teams as opposed to amateur teams. Athletes sometimes participate in regional competitions with fewer elite and professional teams in order to gain experience and game practice. In that case, they earn fewer points even with the same final placement. That is, there are more ranking points to gain at national compared to regional tournaments. For example, finishing first at a regional competition will earn a player only 35 points. There are many factors for the differences in the rankings of players in the same team. For instance, athletes sometimes compete at one or several competitions with a different partner, e.g., in case their standard partner was injured. All ranking points are calculated at the end of the season.

For the purpose of our research, the best eight tournament results were considered, and a ranking list of the best female and male teams and individual players was created. For this research, only the individual ranking points and performance level was considered. Lastly, the participants ranked their satisfaction with the team’s success from 0 to 100%.

### Statistical Analysis

The statistical analysis was in line with the three primary objectives of the study. First, descriptive statistics of the individual beach volleyball athletes’ personality and emotional regulation were calculated. At this point we assessed gender differences by means of an analysis of variance (one-factor ANOVA) which revealed significant differences between both genders for warmth, *F*(1,80) = 7.78, *p* = 0.01, $\eta_{\text{p}}^{2}$ = 0.09, emotional stability, *F*(1,80) = 7.13, *p* = 0.01, $\eta_{\text{p}}^{2}$ = 0.08, dominance, *F*(1,80) = 6.36, *p* = 0.01, $\eta_{\text{p}}^{2}$ = 0.07, sensitivity, *F*(1,80) = 12.33, *p* = 0.001, $\eta_{\text{p}}^{2}$ = 0.13, apprehension, *F*(1,80) = 13.81, *p* < 0.001, $\eta_{\text{p}}^{2}$ = 0.15, and conscientiousness, *F*(1,80) = 4.91, *p* = 0.03, $\eta_{\text{p}}^{2}$ = 0.06. In contrast to the results by Graser et al. (2012), no significant differences between male and female participants’ emotional regulation styles were found within our sample. In order to control for gender differences, gender was entered as covariate for further analysis.

Next, the values were compared to the norm using multiple one-sample *t*-tests. Here, the analysis was adjusted for each questionnaire by Bonferroni correction in order to prevent the accumulation of type 1 errors (Hervé, 2007). *A priori* power analysis (*d* = 0.3, α = 0.05, power = 0.80) revealed a required sample size of *N* = 71.

The second goal of the study was to examine the relationship between personality, emotion regulation style, performance, and team satisfaction. Therefore, partial Pearson correlation was calculated (*a priori* power analysis: *N* = 61 for *d* = 0.35, α = 0.05, power = 0.80).

In order to gather information on similarities or differences in personality traits of both team members, repeated measures MANCOVA analysis was applied (study goal 3). Here, the athlete’s number of the team (athlete A or athlete B) was entered as within-subject factor, gender as covariate, and the scales of the questionnaires as dependent variables. For this analysis, a sample size of 34 was required (*d* = 0.3, α = 0.05, power = 0.80). All the cases with some missing data were excluded and the significance level was set at α = 0.05. All the statistical testing was done with IBM SPSS Statistics Version 27; power analyses were conducted with G^∗^Power 3.1.9.2.

## Results

### Personality of German Beach Volleyball Players

The elite beach volleyball players rated their overall satisfaction with their team’s success at an average of 75.91% (*SD* = 22.27). The mean performance level was 2088.26 (*SD* = 2020.28), which represented approximately 209 national ranking points. The mean values of the personality questionnaires and the emotion regulation style are displayed in Table 1 for all the players. The personality traits of agreeableness and openness to experience were the most reported; neuroticism the least. Compared to the norm (see Rammstedt and John, 2007: ‘German population, both genders, all education levels, 18–35 years’), our sample revealed lower values for extraversion, *t*(81) = −10.19, *p* < 0.001, for agreeableness, *t*(81) = −3.31, *p* = 0.001, and for conscientiousness, *t*(81) = −7.87, *p* < 0.001, and higher values for neuroticism, *t*(81) = 8.35, *p* < 0.001.

**TABLE 1:** Mean values (and standard deviations) of the personality questionnaires and emotion regulation style of elite beach volleyball players.

	Personality Factors (PASK-5)							
	Warmth	Reasoning	Emotional stability	Dominance	Liveliness	Rule-consciousness	Social boldness	Sensitivity

All (*N* = 82)	6.49 (1.70)	7.21 (0.98)	6.49 (1.56)	5.01 (1.43)	6.12 (1.56)	5.82 (1.46)	5.99 (1.42)	5.46 (1.42)
Male (*N* = 46)	6.04 (1.87)	7.23 (0.90)	6.88 (1.47)	5.35 (1.57)	6.07 (1.67)	5.63 (1.53)	6.15 (1.44)	5.00 (1.29)
Female (*N* = 36)	7.06 (1.26)	7.18 (1.10)	5.99 (1.56)	4.57 (1.11)	6.19 (1.44)	6.07 (1.35)	5.79 (1.39)	6.04 (1.39)

	**Vigilance**	**Abstractedness**	**Privateness**	**Apprehension**	**Openness to change**	**Self-reliance**	**Perfectionism**	**Tension**

All (*N* = 82)	5.42 (1.44)	4.45 (1.57)	5.60 (1.52)	4.12 (1.57)	5.51 (1.65)	5.78 (1.67)	6.29 (1.51)	5.24 (0.77)
Male (*N* = 46)	5.70 (1.59)	4.27 (1.56)	5.62 (1.48)	3.59 (1.22)	5.42 (1.73)	5.96 (1.68)	6.45 (1.48)	5.35 (0.85)
Female (*N* = 36)	5.07 (1.16)	4.68 (1.56)	5.57 (1.59)	4.79 (1.71)	5.61 (1.57)	5.56 (1.66)	6.08 (1.53)	5.11 (0.64)

	**Big Five Personality Traits (BFI-10)**							
	**Extraversion**	**Neuroticisms**	**Openness to experience**	**Conscientiousness**	**Agreeableness**			

All (*N* = 82)	3.23 (0.47)	2.94 (0.55)	3.42 (0.72)	3.35 (0.67)	3.16 (0.64)			
Male (*N* = 46)	3.14 (0.40)	2.99 (0.52)	3.46 (0.79)	3.49 (0.59)	3.21 (0.65)			
Female (*N* = 36)	3.33 (0.52)	2.88 (0.59)	3.38 (0.64)	3.17 (0.73)	3.11 (0.63)			

	**Affective Style (ASQ)**							
	**Concealing**	**Adjusting**	**Tolerating**					

All (*N* = 82)	3.09 (0.71)	3.19 (0.65)	3.57 (0.59)					
Male (*N* = 46)	3.16 (0.69)	3.18 (0.71)	3.64 (0.57)					
Female (*N* = 36)	3.00 (0.72)	3.21 (0.56)	3.48 (0.62)					

For the personality factor questionnaire, reasoning, warmth, emotional stability, and perfectionism were the most definite adjectives described for the elite beach volleyball athletes. On the contrary, apprehension and abstractedness were present in fewer instances. Brandstätter (2009) presented norm values for different fields of application, including values for research purposes in his manual. Our sample exhibited higher values in liveliness, *t*(81) = 7.65, *p* < 0.001, tension, *t*(81) = 12.26, *p* < 0.001, openness to change, *t*(81) = 5.52, *p* < 0.001, reasoning, *t*(81) = 7.42, *p* < 0.001, emotional stability, *t*(81) = 3.99, *p* < 0.001, privateness, *t*(81) = 6.55, *p* < 0.001, and lower values in rule-conscientiousness, *t*(81) = −4.19, *p* < 0.001, compared to the norm.

The use of ASQ allowed us to analyze emotion regulation styles and the concrete characteristics of the respondents. Elite beach volleyball players showed high regulation toward their emotions. However, only marginal differences between concealing, adjusting, and tolerating were observed and this was in conformity with the estimated norm values by Graser et al. (2012) (all *p* > 0.02).

### Relationship Between Personality and Performance

As the second objective of the study, the relationship between personality traits, emotional regulation styles, performance, and overall satisfaction was examined. Here, no significant relationship between performance level and personality and emotional regulation of the individual beach volleyball athletes were found^4^. In fact, the overall satisfaction was not related to any of the examined characteristics (all *p* > 0.05; see Table 2). However, we found significant relationships between the questionnaires, which are recorded in Table 3.

**TABLE 2:** Relationship between elite beach volleyball players’ personality, performance, and satisfaction.

*N* = 82	Performance *r* (*p*)	Satisfaction *r* (*p*)
**Personality Factors (PASK-5)**		
Warmth	0.00 (1.00)	−0.07 (0.53)
Reasoning	−0.10 (0.37)	−0.01 (0.92)
Emotional stability	0.21 (0.07)	0.10 (0.37)
Dominance	0.13 (0.24)	−0.16 (0.17)
Liveliness	−0.06 (0.58)	−0.05 (0.64)
Rule-conscientiousness	0.09 (0.45)	−0.00 (0.98)
Social boldness	0.08 (0.49)	0.00 (1.00)
Sensitivity	−0.05 (0.67)	0.09 (0.42)
Vigilance	−0.09 (0.45)	0.14 (0.20)
Abstractedness	−0.03 (0.78)	0.07 (0.53)
Privateness	0.05 (0.64)	−0.10 (0.40)
Apprehension	−0.03 (0.81)	−0.12 (0.29)
Openness to change	−0.02 (0.84)	0.11 (0.34)
Self-reliance	0.16 (0.16)	0.06 (0.61)
Perfectionism	0.16 (0.15)	−0.11 (0.33)
Tension	−0.06 (0.57)	−0.14 (0.23)
**Big Five Personality Traits (BFI-10)**		
Extraversion	0.13 (0.24)	0.10 (0.37)
Neuroticism	−0.18 (0.10)	0.03 (0.77)
Openness to experience	0.03 (0.82)	−0.06 (0.61)
Conscientiousness	−0.09 (0.45)	−0.13 (0.24)
Agreeableness	0.08 (0.51)	−0.18 (0.12)
**Affective Style (ASQ)**		
Concealing	0.03 (0.81)	−0.20 (0.86)
Adjusting	0.10 (0.40)	0.09 (0.44)
Tolerating	0.06 (0.63)	0.05 (0.67)

**TABLE 3:** Pearson correlation between the Big Five personality traits, personality adjective scales and emotion regulation styles of elite beach volleyball players.

*N* = 82		PE	S	W	R	ES	D	L	RC	SB	S	V	AB	PR	AP	OC	SR	PF	T	E	N	OE	C	AG	CL	AD	TO
Performance PE	*r*	1	0.16	0.00	–0.10	0.21	0.13	–0.06	0.08	0.08	–0.05	–0.09	–0.03	0.05	–0.03	–0.02	0.16	0.16	–0.06	0.13	–0.18	0.03	–0.09	0.08	0.03	0.09	0.05
	*p*		0.15	1.00	0.37	0.07	0.24	0.57	0.45	0.49	0.67	0.45	0.78	0.63	0.81	0.84	0.16	0.15	0.57	0.23	0.10	0.82	0.45	0.51	0.81	0.40	0.63
Satisfaction S	*r*		1	–0.07	–0.01	0.10	–0.16	–0.05	0.00	0.00	0.09	0.14	0.07	–0.10	–0.12	0.11	0.06	–0.11	–0.14	0.10	0.03	–0.06	–0.13	–0.17	–0.02	0.09	0.05
	*p*			0.53	0.92	0.37	0.17	0.64	0.98	1.00	0.42	0.20	0.53	0.40	0.29	0.34	0.61	0.33	0.23	0.37	0.77	0.61	0.24	0.12	0.86	0.44	0.67
Warmth W	*r*			1	–0.05	0.07	0.01	0.47	−*0.37*	*0.46*	0.00	−*0.40*	*0.29*	–0.21	−*0.31*	*0.31*	–0.07	–0.06	0.12	0.05	0.06	0.12	–0.15	*0.22*	–0.09	0.05	0.19
	*p*				0.65	0.54	0.96	*0.00*	*0.00*	*0.00*	0.97	*0.00*	*0.01*	0.06	*0.01*	*0.00*	0.54	0.62	0.29	0.65	0.62	0.28	0.19	*0.04*	0.45	0.64	0.10
Reasoning R	*r*				1	0.20	0.12	0.04	0.10	0.20	0.01	0.08	−*0.28*	*0.34*	−*0.23*	0.04	0.21	*0.35*	−*0.29*	–0.18	–0.17	0.03	–0.14	–0.14	0.12	0.19	–0.09
	*p*					0.08	0.31	0.73	0.37	0.07	0.95	0.50	*0.01*	*0.00*	*0.04*	0.71	0.06	*0.00*	*0.01*	0.12	0.14	0.81	0.20	0.21	0.28	0.09	0.40
Emotional stability ES	*r*					1	0.02	0.04	–0.18	*0.25*	−*0.23*	–0.14	–0.08	–0.02	−*0.32*	0.16	0.09	*0.30*	–0.11	0.14	0.01	–0.15	–0.17	0.05	0.16	*0.30*	0.12
	*p*						0.83	0.73	0.11	*0.02*	*0.04*	0.20	0.50	0.87	*0.00*	0.14	0.43	*0.01*	0.33	0.20	0.93	0.17	0.13	0.67	0.14	*0.01*	0.27
Dominance D	*r*						1	*0.32*	0.05	*0.35*	−*0.28*	*0.23*	0.01	−*0.32*	–0.06	0.12	*0.32*	–0.05	0.06	–0.18	0.10	0.01	–0.14	0.08	–0.17	–0.13	–0.01
	*p*							*0.00*	0.67	*0.00*	*0.01*	*0.04*	0.94	*0.00*	0.59	0.28	*0.00*	0.65	0.57	0.11	0.39	0.93	0.20	0.46	0.12	0.26	0.93
Liveliness L	*r*							1	–0.20	*0.72*	−*0.28*	–0.05	*0.29*	−*0.44*	–0.18	*0.44*	0.02	–0.11	*0.27*	–0.12	0.00	0.18	–0.21	*0.24*	–0.19	–0.01	*0.31*
	*p*								0.07	*0.00*	*0.01*	0.68	*0.01*	*0.00*	0.11	*0.00*	0.87	0.31	*0.01*	0.30	0.98	0.12	0.06	*0.03*	0.09	0.96	*0.01*
Rule-consciousness RC	*r*								1	−*0.34*	0.15	0.14	−*0.44*	*0.43*	*0.35*	−*0.39*	0.14	*0.33*	–0.17	–0.09	–0.16	–0.14	0.03	–0.01	0.09	–0.01	–0.07
	*p*									*0.00*	0.19	0.22	*0.00*	*0.00*	*0.00*	*0.00*	0.21	*0.00*	0.12	0.41	0.15	0.22	0.76	0.90	0.42	0.92	0.52
Social boldness SB	*r*									1	−*0.33*	0.03	*0.29*	−*0.39*	−*0.41*	*0.48*	0.19	0.01	0.17	–0.07	–0.16	0.09	–0.22	*0.22*	–0.13	0.06	*0.26*
	*p*										*0.00*	0.80	*0.01*	*0.00*	*0.00*	*0.00*	0.08	0.94	0.12	0.55	0.16	0.43	0.05	*0.04*	0.25	0.60	*0.02*
Sensitivity S	*r*										1	−*0.24*	–0.05	*0.40*	0.08	−*0.28*	−*0.26*	0.07	0.01	0.04	0.02	0.03	0.07	0.02	–0.13	–0.16	–0.02
	*p*											*0.03*	0.66	*0.00*	0.47	*0.01*	*0.02*	0.53	0.95	0.75	0.89	0.80	0.52	0.86	0.26	0.15	0.85
Vigilance V	*r*											1	–0.11	–0.14	0.14	–0.13	*0.34*	–0.04	–0.03	–0.14	–0.11	–0.09	−*0.27*	–0.21	–0.04	−*0.28*	–0.04
	*p*												0.34	0.23	0.20	0.24	*0.00*	0.72	0.79	0.21	0.33	0.40	*0.02*	0.06	0.73	*0.01*	0.71
Abstractedness AB	*r*												1	−*0.36*	–0.07	*0.32*	–0.16	−*0.47*	*0.25*	–0.03	–0.01	0.19	–0.07	0.01	−*0.29*	–0.18	0.21
	*p*													*0.00*	0.54	*0.00*	0.16	*0.00*	*0.02*	0.77	0.92	0.10	0.52	0.96	*0.01*	0.12	0.06
Privateness PR	*r*													1	0.07	−*0.43*	0.02	*0.44*	–0.15	–0.07	–0.17	–0.20	–0.04	–0.11	0.15	0.16	−*0.23*
	*p*														0.53	*0.00*	0.87	*0.00*	0.18	0.54	0.13	0.07	0.75	0.34	0.17	0.15	*0.04*
Apprehension AP	*r*														1	−*0.30*	0.01	–0.05	–0.05	–0.10	0.02	0.00	0.08	–0.12	0.15	–0.07	−*0.23*
	*p*															*0.01*	0.91	0.65	0.63	0.36	0.88	0.98	0.48	0.29	0.20	0.55	*0.04*
Openness to change OC	*r*															1	0.16	–0.20	–0.02	–0.14	0.04	–0.08	–0.16	0.02	0.00	*0.24*	0.09
	*p*																0.15	0.07	0.88	0.21	0.74	0.48	0.16	0.87	0.98	*0.03*	0.42
Self-reliance SR	*r*																1	0.14	–0.11	−*0.28*	–0.16	–0.18	−*0.37*	–0.07	0.16	0.16	–0.02
	*p*																	0.21	0.31	*0.01*	0.15	0.12	*0.00*	0.55	0.15	0.16	0.87
Perfectionism PF	*r*																	1	−*0.32*	0.03	−*0.24*	–0.08	–0.03	–0.09	0.22	*0.30*	–0.04
	*p*																		*0.00*	0.80	*0.03*	0.46	0.78	0.40	0.05	*0.01*	0.71
Tension T	*r*																		1	–0.09	–0.05	–0.01	–0.02	0.12	–0.21	−*0.25*	*0.34*
	*p*																			0.41	0.68	0.95	0.83	0.29	0.06	*0.02*	*0.00*
Extraversion E	*r*																			1	0.16	–0.04	0.18	0.00	–0.03	–0.04	–0.03
	*p*																				0.14	0.75	0.11	0.98	0.77	0.70	0.81
Neuroticism N	*r*																				1	–0.07	0.16	–0.08	0.12	0.12	–0.12
	*p*																					0.52	0.14	0.50	0.27	0.29	0.27
Openness to experience OE	*r*																					1	0.18	0.20	–0.18	–0.10	0.15
	*p*																						0.11	0.08	0.12	0.37	0.19
Conscientiousness C	*r*																						1	0.06	0.13	0.04	–0.10
	*p*																							0.61	0.25	0.70	0.37
Agreeableness AG	*r*																							1	–0.14	–0.05	0.08
	*p*																								0.22	0.65	0.45
Concealing CL	*r*																								1	*0.62*	–0.21
	*p*																									*0.00*	0.06
Adjusting AD	*r*																									1	–0.06
	*p*																										0.62
Tolerating TO	*r*																										1
	*p*																										

*Italics represent significant correlations.*

### Personality Differences Within Beach Volleyball Teams

The third objective of the study was to determine whether the elite beach volleyball players within a team share the same personality characteristics or if team members differ in certain personality traits. The data from 40^5^ elite beach volleyball teams was compared to assess that. As the results of the mean differences between the personality traits, personality factors, and the emotion regulation style already suggest, only marginal differences in personality traits between the beach volleyball players of the same team exist (see Table 4). Moreover, the repeated measures MANCOVA showed no significant differences between both members of a team with regard to those dependent variables, *F*(23,16) = 1.22, *p* = 0.35. Therefore, it can be assumed that the elite beach volleyball teams are composed of rather similar than complementary personalities.

**TABLE 4:** Intra-team differences of personality and emotion regulation for elite beach volleyball teams (*N* = 40).

	Personality Factors (PASK-5)							
	Warmth	Reasoning	Emotional stability	Dominance	Liveliness	Rule-consciousness	Social boldness	Sensitivity
*M* (*SD*)	1.88 (1.33)	1.19 (0.86)	1.71 (1.04)	1.45 (1.15)	1.70 (1.31)	1.64 (1.32)	1.58 (1.28)	1.40 (1.37)

	**Vigilance**	**Abstractedness**	**Privateness**	**Apprehension**	**Openness to change**	**Self-reliance**	**Perfectionism**	**Tension**

*M* (*SD*)	1.64 (1.19)	1.70 (1.33)	1.76 (1.22)	1.73 (1.04)	2.01 (1.38)	1.81 (1.38)	1.44 (1.14)	0.89 (0.74)

	**Big Five Personality Traits (BFI-10)**										
	**Extraversion**	**Neuroticisms**	**Openness to experience**	**Conscientiousness**	**Agreeableness**			

*M* (*SD*)	0.48 (0.41)	0.53 (0.42)	0.84 (0.62)	0.65 (0.53)	0.70 (0.65)			

	**Affective Style (ASQ)**							
	**Concealing**	**Adjusting**	**Tolerating**					

*M* (*SD*)	0.70 (0.51)	0.73 (0.53)	0.71 (0.50)					

## Discussion

The current study was designed to assess the personality profiles of elite German beach volleyball players. It turned out that the personality trait of neuroticism was present more compared to the norm. However, extraversion, agreeableness, and conscientiousness were found less distinct. The personality profile of the players further revealed enhanced characteristics of liveliness, tension, openness to change, privateness, emotional stability, as well as reasoning, and reduced rule-conscientiousness. Moreover, the participating team members possessed well-established emotion regulation styles. In general, beach volleyball players can be characterized as spontaneous and lively. They value friendly social relationships, do not worry much, and are rather stable in their cognitive assessment (Brandstätter, 2009). They are focused as well as goal-oriented, and show high social competence (Allen et al., 2013). They also apply different emotion regulation styles that help them to deal with withdrawn affection and this leads them to remain focused on the match.

Even though, the study did not control for situational selection of the regulation strategies, it can be assumed that contextual variables have an influence on the selection of the emotion regulation strategies. In competitions, it is often beneficial to hide and conceal one’s own emotions in order to prevent an opponent from capitalizing on them, e.g., insecurity (Gross and John, 2003; Graser et al., 2012). Additionally, reappraisal of situations and adjusting own reactions to negative events (e.g., own mistakes and provoking opponent) helps to remain calm and positive (Mauss et al., 2007). Lastly, having higher tolerance toward one’s own emotions and accepting affects as they come, enhances an athlete’s wellbeing and the ability to deal with stress (Campbell-Sills et al., 2006; Kashdan and Steger, 2006). Yet, previous results of a study establishing a relationship between personality traits – especially extraversion, conscientiousness, agreeableness – and performance (Allen et al., 2013) could not be confirmed as no significant relationship between personality and emotion regulation and performance were found. In our study, we only observed individual emotion regulation strategies. However, in team sports, it can be beneficial and, in fact, necessary to regulate one’s teammate’s emotions as well, e.g., to help a teammate cope with his/her emotions or to prevent a negative impact of such emotions on one’s own emotions or performance (Campo et al., 2017). This interpersonal emotion regulation approach can enable better communication, collaborative work and ultimately, better team functioning (Brandwein et al., 2021). There is further scope to examine interpersonal emotion regulation strategies within beach volleyball teams.

Furthermore, we analyzed the relationship between personality and emotion regulation styles with performance and satisfaction. No significant relationship between performance, satisfaction, and personality was found. Similarly, one study by Waleriańczyk and Stolarski (2021) found no relationship between the Big-Five personality traits and race performance. However, in this study, the authors controlled for perfectionism, which showed a positive correlation to performance as well as the personality trait of conscientiousness. Gyomber et al. (2016) controlled for further psychological variables, like coping mechanisms and anxiety as personality traits in relation to performance. They found that athletes that can cope with pressure and are able to overcome it to exhibit peak performance in demanding situations, have greater performance outputs. Furthermore, neuroticism was negatively related to performance. As previous research has indicated (e.g., Halfhill et al., 2005; Woodman et al., 2010; Allen et al., 2013; Piepiora, 2021), personality and other psychological variables like emotion regulation, perfectionism, or coping mechanisms, indeed contribute to the performance of an athlete and future research should go beyond the Big Five personality traits to include other psychological variables (Roberts and Woodman, 2017).

One possible explanation for our contradictory findings might be that the performance was compared to the earned ranking points of the players. Players who won a higher number of matches and competed at more tournaments achieved higher rankings than athletes who either competed at fewer tournaments and/or did not win as often. Consequently, the actual performance level might not have been represented adequately by ranking points, as a team can, for example, win a match despite playing poorly (subjectively rated poor performance) or lose a match despite playing well (subjectively rated good performance). Therefore, future research should consider subjective evaluations from athletes, coaches, etc. about performance as a part of the measurement.

Another methodological problem concerns the nature of sport performance. While personality seems a stable set of characteristics (Seligman et al., 2005), performance and success are season or competition dependent. However, during and across seasons or competitions (or even in matches), performance can be stable (e.g., several top-5 placements following each other, participation in highest level competitions for years) as well as unstable (e.g., variations in ranking, relegation and ascent), which is reflected in ranking points. It is, therefore, necessary to control for performance trends via longitudinal or time-series analyses. Additionally, it has to be examined whether personality as a stable characteristic contributes to immediate performance or only functions as a foundation for achieving peak performance. Nevertheless, it might also be the case that personality is not a valid predictor for success in beach volleyball. Aspects like athleticism, technique, tactic, self regulation (Klatt and Noël, 2020) and cognitive abilities, (e.g., decision making; Noël et al., 2016; Klatt and Smeeton, 2020, or gaze behavior, Hüttermann et al., 2018) might be of higher relevance within this sport so that these factors should also be considered in more detail in future research. In this relation it was recently found that, among others, at least in referee teams in different sports, not only individual cognitive abilities play a role, but that the ability to coordinate the behavior of different team members is important for guaranteeing a high level of team performance (cf. Fasold et al., 2019; Fasold et al., 2021; Klatt et al., 2021).

Another objective of the study was to examine whether personality characteristics of the players were similar within dyadic teams. Within our sample, only marginal intra-team and no overall significant differences between the personalities of both team members were found. *Post hoc* analyses (G^∗^Power 3.1.92) revealed a test statistic power (1 – β) of 0.94. Therefore, we can conclude that German beach volleyball teams are usually composed of players sharing similar personality characteristics. Comparable results were found by Jackson et al. (2010, 2011) and Cameron et al. (2012), however, the overall results still remain inconclusive as König-Görögh et al. (2017) and Álvarez-Kurogi et al. (2019) found differences in personality of various positions within handball and futsal teams.

Similar personalities within dyadic teams were associated with fewer conflicts and better intra-team relationships (Jackson et al., 2010). However, some teams might be able to benefit from different personality characteristics (Mohammed and Angell, 2003; Beebe and Masterson, 2010; Gilley et al., 2010). Here, factors like similar goal setting, physical condition, motivation, and different as wells as complementary expertise are considered for team formation (Memmert et al., 2015; Budak et al., 2018). Therefore, both similarity and diversity within teams can be vital, depending on how diverse the requirements are for the different player positions within a sport or team (cf. Fasold et al., 2020). However, it is still unclear whether similar personalities in team sports are beneficial for performance and success. Future studies should, thus, include measurements of cohesion and relationship quality, conflict management, and team selection processes. As conflicts and relationship quality were influenced by personality, it could be that the beneficial effect of cohesion on success (e.g., see Carron et al., 2002) might be explained by similar personalities.

As no information about the team or player selection process was gathered, we cannot verify whether personality was at all considered during the beach volleyball team formation phase. Furthermore, it could be that the athletes themselves were either running the team selection process or had a great influence on it. As a result, player selection might unconsciously be influenced by preferences for a player sharing similar beliefs, norms, values, and personality traits.

There are some limitations and considerations for future research that need to be acknowledged. Within our study, we used self-reports to gain information about personality, emotion regulation, success/performance, and satisfaction of athletes within a beach volleyball team. We chose this approach because it minimized the time commitment for participants and allowed them to participate in the study despite a demanding time schedule during their season. Furthermore, our design did not allow for a cause-effect relationship. In order to gain a better understanding of personality and emotion regulation as predictors for success and satisfaction, an experimental design, which is more time consuming and requires an interference in training (or alternatively an additionally scheduled measurement), would be necessary.

## Conclusion

The current study investigated the personality and emotion regulation styles of elite beach volleyball players. Within these teams, personality characteristics of the athletes were rather similar, and it can be assumed that teams generally consist of athletes who share certain personality traits and values. However, personality and emotion regulation were not related to the actual performance level as per our findings. Other motor (e.g., athleticism and technique) and psychological (e.g., cohesion and motivation) factors should be taken into account in future in order to find factors leading to peak performance. Additionally, as our research was only conducted in Germany, information about the personality of beach volleyball players from other countries, cultures, or ethnicities may also need to be studied to verify our results.

Our findings are of great interest to sports psychology practitioners, coaches, and scouts. In order to work with high-performance athletes, knowledge about an individual’s personality might help in creating a functional working-relationship. Existing research has shown that a coach-athlete relationship is more beneficial when both share similar personality characteristics (Jackson et al., 2011). Therefore, our findings might help coaches to better understand their players’ personality, beliefs as well as values, and consequently choose a suitable coaching-style, which helps prevent and/or solve conflicts. Coaches should also encourage their players to explore their own and their teammate’s personality in order to gain better understanding of their team dynamics. Applied sports psychologists can support this process and provide knowledge about conflict resolution strategies and encourage self-esteem and self-acceptance.

Sports psychologists can also help in developing and strengthening emotion regulation strategies. As our sample showed, emotion regulation strategies were already well established. However, young athletes might not have developed enough strategies to deal with their own emotions. In summary, our research emphasizes on the importance of recognizing (beach volleyball) athletes’ personalities and emotion regulation strategies in order to create competitive dyads in team sports.

## Data Availability Statement

The raw data supporting the conclusions of this article will be made available by the second author, without undue reservation, to any qualified researcher.

## Ethics Statement

The studies involving human participants were reviewed and approved by German Sport University Cologne. The patients/participants provided their written informed consent to participate in this study.

## Author Contributions

SK and BN developed the study concept and contributed to the design. SW collected the data. L-MR analyzed the data and wrote the first draft of the manuscript. All authors helped to edit and revised the manuscript and approved the final submitted version of the manuscript.

## Conflict of Interest

The authors declare that the research was conducted in the absence of any commercial or financial relationships that could be construed as a potential conflict of interest.

## Publisher’s Note

All claims expressed in this article are solely those of the authors and do not necessarily represent those of their affiliated organizations, or those of the publisher, the editors and the reviewers. Any product that may be evaluated in this article, or claim that may be made by its manufacturer, is not guaranteed or endorsed by the publisher.

## Footnotes

1
https://www.soscisurvey.de/

2 “A level tournaments” are the highest ranked regional competitions. The ranking system is structured as the following (from lowest to highest): D level, C level, B level, A level (all regional), category 1, category 1+, and category 2 (all national). For further information on the regional level ranking see https://wp.beachvolleyball.nrw/wp-content/uploads/2021/07/Durchfuehrungsbestimmungen-WVV-Beachserie-2021_14.07.2021-Website.pdf, on national level ranking see https://www.volleyball-verband.de/?proxy=redaktion/Dokumente/Satzung-Ordnungen/Beach-Volleyball-Ordnung/2021/20210602_BVO_Anlage-F_Durchfuehrungsbestimmungen_LV_2021_V18.pdf; Both last accessed on 7th of September 2021.

3 https://beach.volleyball-verband.de; Last accessed on 22nd of October 2020.

4 As performance level was compared to individual ranking points, no analysis examining personality differences and team performance were presented.

5 From the 82 athletes, two athletes had to be excluded from intra-teams comparisons, as their partner did not provide complete data. Therefore, only 40 teams were included for the third objective of the study.
